# When Innovation Leaves People Behind: Reframing Accountability in Commercial Digital Health

**DOI:** 10.2196/100654

**Published:** 2026-09-17

**Authors:** Hajira Dambha-Miller, Lucy Smith, Lysanne Veerle Michels

**Affiliations:** 1 Primary Care Research Centre, University of Southampton, University Road, Southampton, England, SO17 1BJ, United Kingdom, 44 (0)23 8059 ext 5000

**Keywords:** health care, public health, digital health, inequity, bias

## Abstract

Digital technologies are increasingly integrated across health care systems; however, accumulating evidence shows that these tools often perform unevenly across population groups. Biases in AI-enabled tools can reinforce existing health inequities, particularly when systems are developed and validated using datasets that exclude underserved populations. Studies demonstrate systematic underestimation of illness severity, diagnostic inaccuracies, and measurement bias affecting ethnic minority groups, rural populations, people with disabilities, people experiencing homelessness, and other groups with limited access to health care. These disparities highlight structural vulnerabilities in the data that inform machine learning systems, where socially patterned access to care shapes what is recorded and therefore learned. Commercial adoption patterns further exacerbate inequities due to socioeconomic gradients in digital engagement and a lack of accountability measures for adherence to regulatory frameworks within National Health Service procurement processes. In this Viewpoint, we argue that ensuring accountability in digital health care requires transparent reporting of subgroup performance, representative development of datasets, and ongoing monitoring of distributional impacts. Achieving equitable and reliable digital health care demands regulatory and methodological standards that prioritize fairness and generalizability within the commercialization sector and are monitored across the pre- and postdeployment life cycle.

## Introduction

Digital technologies are now embedded within routine health care delivery across high-income and middle-income settings. AI-enabled diagnostic systems, predictive risk models, and remote monitoring platforms are integrated into primary care, hospital medicine, and community services [1]. These are frequently justified as necessary responses to workforce shortages, demographic change, and fiscal constraints. However, accumulating empirical evidence indicates that digital health technologies do not perform uniformly across patient populations and that differential performance has direct clinical consequences [2]. The central issue is not solely unequal access to innovation but also the lack of consideration of equity in product design. Clinical tools need to demonstrate reliability across the populations in which they are deployed, and generalizability remains a core requirement of safe medical practice. When performance varies systematically by ethnicity, socioeconomic position, or geography, this amplifies inequity that is already present in both the health system and wider society [3].

While concerns about algorithm bias have received increasing attention within the digital health care literature, the role of commercial developers, regulators, and procurers within the current health system as drivers of inequity has been largely overlooked. Throughout this Viewpoint, we use the term “commercial digital health” to refer to digital technologies developed and marketed by private sector organizations for deployment within health care systems. These include AI-enabled clinical decision support systems, predictive risk algorithms, digital triage platforms, remote observation technologies, and software as a medical device. In contrast to digital systems developed internally by health care organizations for local use, where governance, monitoring, and oversight structures are often embedded within existing organizational frameworks, commercial products are frequently deployed across multiple organizations and populations. However, the underlying training data, validation processes, and assessment of metrics may remain proprietary. This creates unique accountability challenges, particularly when procurement decisions are made without access to detailed evidence on subgroup performance, the representativeness of development datasets, or ongoing monitoring arrangements.

Recent frameworks by Liu et al [4] and Chin et al [5] have illustrated the importance of representative datasets, subgroup performance assessment, fairness monitoring, and fair outcomes across the AI life cycle. However, these approaches have primarily focused on algorithm development and performance. We argue that inequity in digital health is also shaped by commercial incentives, procurement decisions, and patterns of digital exclusion that determine who is represented in datasets and who ultimately benefits from innovation. This Viewpoint therefore broadens existing AI fairness frameworks by proposing an accountability model that spans the full commercial digital health life cycle, from product development and procurement through implementation and postmarket monitoring. We put forth the case that evidence of equitable performance should become a prerequisite for the procurement, implementation, and continued use of commercial digital health technologies. Just as safety and effectiveness are routinely assessed before adoption, demonstrable impartiality across relevant population groups should be considered a core requirement of trustworthy health innovation.

## Existing Understanding of Mechanisms of Algorithmic Bias

Inequities associated with digital health technologies arise through 4 key mechanisms that should not be considered interchangeable. First, measurement bias can occur when digital tools perform differently across populations. In a cohort of 10,789 patients, Sjoding et al [6] reported that occult hypoxemia occurred in 11.7% of Black patients compared with 3.6% of White patients when assessed by pulse oximetry, a device ubiquitous in acute and community care. Pfohl et al [7] highlight that predictive algorithms often experience performance degradation when deployed in populations that differ from those used during model development. This phenomenon, known as distribution shift, can disproportionately affect underrepresented patient groups and increase health care inequities.

Second, dataset bias can occur when training data used for machine learning software do not represent the population in which technologies are to be deployed. Obermeyer et al [8] examined a commercial risk prediction algorithm affecting more than 200 million individuals and found that it systematically underestimated illness severity among Black patients because it relied on health care expenditure as a proxy for need. As expenditure reflects access to services rather than biological burden, the model encoded structural inequity into resource allocation decisions; correcting the bias increased the proportion of Black patients eligible for additional care from 17.7% to 46.5%. This can be influenced by the third mechanism: algorithmic bias may emerge when models learn patterns that reflect existing inequities within health care systems. If underserved populations do not have equitable access to health care services, less data reflecting their actual health care needs will be available to train models. Seyyed-Kalantari et al [9] demonstrated underdiagnosis bias in deep learning systems applied to chest radiographs among underserved populations despite comparable aggregate performance. In dermatology, limited representation of darker skin tones in training datasets has been associated with reduced diagnostic reliability in underserved populations [10]. Fourth, digital exclusion can occur when certain populations face barriers to accessing or engaging with digital technologies. This presents fewer opportunities for subgroup analysis to be performed to assess model accuracy across populations—we may only know that the digital tool works for those who are already competent with health technology.

**Figure 1.**
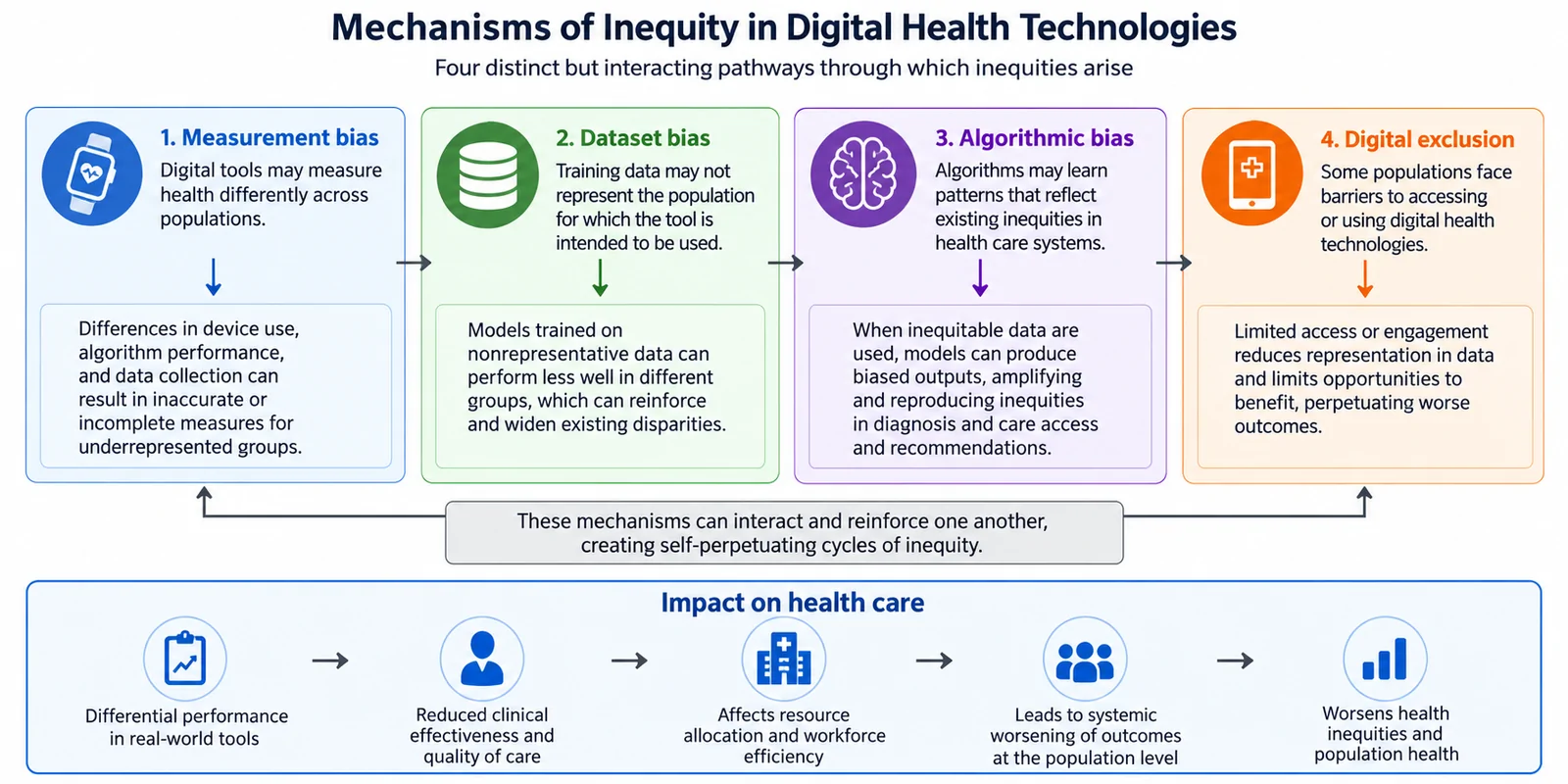
Flowchart describing mechanisms of inequity in digital health technologies.

Although these mechanisms can interact and reinforce one another, they originate from distinct causes and require different approaches to mitigation. These findings differ in mechanisms but converge in implication: differential performance in widely deployed technologies alters clinical classification, escalation, and access to care. Figure 1 visually depicts these 4 mechanisms.

These disparities expose a structural epistemic vulnerability, whereby systemic inequity shapes the data from which AI systems learn. Machine learning systems are trained on historical datasets generated within health systems characterized by socially patterned access, investigation, and documentation. Electronic health records reflect who presents, who is investigated, and whose symptoms are recorded; however, these patterns do not represent true clinical need. Rather, they reflect the wider socioeconomic and cultural barriers to help-seeking behaviors or geographical disparities in service provision. For example, people with disabilities, individuals from ethnic minority groups, rural populations, people who are experiencing homelessness, and people from sexual and gender minority groups are frequently documented as having less access to health care [11]. Missingness and sampling bias can propagate through predictive models [12], and aggregate discrimination statistics may obscure clinically significant subgroup variability; a model with high overall discrimination can nonetheless systematically misclassify individuals within specific populations.

The implications are immediate—social injustice becomes encoded as algorithmic bias, and “majority” phenotypes and populations are prioritized in technological development. Algorithms influence triage thresholds, referral decisions, and allocation of scarce resources. Elevated false-negative rates in certain groups may delay diagnosis and treatment; differential calibration may misrepresent disease burden. In pharmacological and device evaluation, limited representation is recognized as a constraint on external validity. Digital systems, by contrast, may be deployed across regional or national infrastructures, affecting hundreds of thousands of patients without routine disclosure of stratified performance metrics. Rajkomar et al [13] argue that fairness assessment must be integral to clinical machine learning; however, consistent standards for subgroup reporting remain underdeveloped.

## Commercial Accountability for Equitable Digital Health Implementation: A Novel Mechanism of Bias

Rajkomar et al [13] argue that achieving fairness in health care AI extends beyond improving algorithm performance, requiring consideration of how data quality, model development, and implementation influence health equity. This highlights that reducing algorithmic bias requires organizational and clinical governance in addition to technical solutions. Liu et al [4] and Chin et al [5] have highlighted measurement bias, dataset bias, and algorithmic bias as important drivers of inequitable AI performance. We build on this work by arguing that commercial dynamics compound these methodological concerns, bringing to the surface additional mechanisms through which inequities may be reproduced and amplified during implementation, including digital exclusion and commercialization.

The development and refinement of digital health technologies are frequently driven by commercial incentives that prioritize rapid deployment and scalability, potentially at the expense of equitable design and long-term monitoring. For example, AI-enabled self-management tools for serious mental illnesses (SMIs), such as addiction and schizophrenia, lag substantially behind those for mild to moderate mental health conditions in development. Market shapes incentive: in the example of people with mild to moderate mental health conditions, there is less need for clinical governance and products can be sold directly to consumers. In comparison, people with SMI often also experience digital exclusion, fragmented service contact, and poverty. Consequently, the commercial return is smaller, while development and governance requirements are greater. Smith et al [14] argue that commercial AI development is fundamentally influenced by market incentives, meaning that populations generating the largest and most profitable datasets are often prioritized during product development. This raises questions regarding whether commercial development pathways preferentially prioritize populations who are easier to reach, retain, and monetize, potentially reinforcing existing patterns of exclusion and further driving algorithmic bias.

Aside from the example from SMI, the adoption of digital health technologies is socially patterned across diverse settings. A systematic review of 41 studies identified persistent socioeconomic gradients in digital health uptake, with lower engagement among individuals with reduced income, education, and digital literacy [15]. In the United Kingdom, approximately 6% of households lack internet access, with substantially higher exclusion among older and lower-income groups [16]. Iterative model refinement frequently relies on user-generated data; populations most engaged with digital systems therefore disproportionately shape subsequent optimization, rendering representation self-reinforcing and creating a feedback loop in which existing patterns of representation become increasingly entrenched. These dynamics highlight that algorithmic bias is not solely a technical phenomenon but is also shaped by the commercial, social, and structural contexts within which digital technologies are developed and deployed.

Available frameworks addressing AI fairness in health care have primarily focused on algorithm-level bias, including representativeness of training data, model effectiveness across demographic groups, and ongoing fairness monitoring throughout the AI life cycle. While these considerations remain essential, they largely assume that patients can access and engage with AI-enabled services. We argue that this assumption overlooks a critical upstream determinant of equity: market exclusion. For many populations, including older adults, people living with multiple long-term conditions, individuals with lower socioeconomic status, and those with limited digital literacy, inequity may arise before algorithmic interaction occurs. This could lead to an intervention-generated inequity, where only users who have the resources to engage with digital health tools do so, benefiting individuals with greater technological, educational, and socioeconomic resources [17]. This cycle is perpetuated by market needs. Consequently, we propose shifting from a narrow focus on AI fairness to a broader model of AI accessibility and inclusion that considers barriers across the entire commercialization digital care pathway, from access and engagement through to algorithm accuracy and long-term outcomes.

## Commercialization and the Limits of Current AI Governance

The European Union Artificial Intelligence Act [18] and World Health Organization [19] guidance have strengthened expectations for transparency, risk management, and postdeployment monitoring of AI-enabled health care technologies. These frameworks recognize that digital tools are increasingly embedded within routine care and that even modest differences in performance may generate substantial inequities when deployed at scale. However, existing governance approaches focus primarily on the safety, effectiveness, and fairness of algorithms. Less attention has been given to how commercial ownership, proprietary development processes, and procurement practices affect the ability of health care organizations to independently evaluate, monitor, and challenge these technologies once deployed.

The nature of commercial ownership limits health care organizations’ practical ability to verify that these principles have been achieved. National Health Service procurement frameworks, including Digital Technology Assessment Criteria [20] and NHS England’s AI Buyer’s Guide [21], require evidence of safety, effectiveness, and statutory compliance. However, they do not require comprehensive disclosure of proprietary datasets, model training procedures, or algorithm-based architecture, thereby rendering health care organizations largely dependent on evidence generated by manufacturers themselves. This constrains opportunities for independent scrutiny of likely biases, representativeness, and real-world validity, creating an asymmetry of knowledge between developers, health care organizations, regulators, and patients. Narrowing this implementation gap between development and transparent deployment necessitates a shift from regulation frameworks toward stronger mechanisms for developer accountability, independent evaluation, and representative postdeployment observation.

## Reframing Accountability in Commercial Digital Health

### Overview

Accountability in commercial digital health must consequently be reframed as a matter of clinical safety and methodological integrity. Demonstration of overall discrimination or calibration is insufficient where subgroup performance varies in clinically meaningful ways. Transparent reporting of validation metrics stratified by relevant demographic and socioeconomic characteristics should be mandatory, and postimplementation monitoring must assess distributional effects in real-world settings rather than aggregate performance alone. Regulatory approval, health technology assessment, and procurement decisions within publicly funded health systems should require evidence of representativeness in development cohorts and reproducible performance across heterogeneous populations. Digital technologies hold substantial promise for strengthening health systems facing rising multimorbidity and constrained capacity; however, scale amplifies both benefit and harm [20]. Systems that function reliably across diverse populations are more likely to sustain professional trust and regulatory legitimacy.

Existing frameworks have made important contributions by emphasizing representative datasets, fairness assessment, disclosure, and life cycle monitoring of health care algorithms. For example, Chin et al [5] propose guiding principles spanning the full algorithm life cycle, including stakeholder involvement, fairness monitoring, and accountability for outcomes, while Liu et al [4] focus on the importance of representative datasets and the assessment of subgroup performance. However, these frameworks focus primarily on the governance of algorithms themselves. We extend this discussion by examining how commercialization, procurement processes, and digital exclusion influence which populations are represented in technology development and which ultimately benefit from implementation. We therefore argue that commercial accountability should be a complementary component of equitable digital health implementation. We make the following actionable recommendations that offer solutions for reframing accountability in commercial digital health.

### Transparency and Reporting Standards for Commercial Digital Health Technologies

Developers of commercial digital health technologies should be required to publish validated performance metrics before implementation, making this information available to both procurers and regulators. Similar to reporting requirements for other health care technologies, these metrics should include calibration, sensitivity, specificity, false-negative rates, threshold effects, CIs, patterns of missing data, and evidence of performance disparities in real-world use. Developers should also disclose the demographic, socioeconomic, and digital characteristics of the populations represented in development and validation of datasets. This information is essential to determine whether technologies have been developed and validated primarily using data from highly engaged or digitally connected populations and to assess their likely generalizability to underserved groups.

### Equity-Focused Procurement Requirements

Public health care organizations should require commercial vendors to provide evidence demonstrating model performance across relevant demographic and clinical subgroups, including age, sex, ethnicity, socioeconomic status, disability status, and comorbidity burden. Vendors should also disclose key characteristics of development and validation of cohorts, including evidence that populations at risk of digital exclusion were adequately represented. Procurement processes should recognize that commercial development may preferentially target populations that are easier to recruit, more digitally engaged, and commercially attractive. Procurement decisions should therefore explicitly consider whether technologies have been evaluated in underserved populations that are frequently underrepresented in commercial development pathways.

### Ongoing Monitoring of Equity After Implementation

Postimplementation monitoring should be undertaken at predefined time points throughout the deployment life cycle and should include routine performance audits for underserved populations and individuals at risk of digital exclusion. Evaluation should assess not only algorithm performance, referral patterns, and diagnostic outcomes but also technology uptake and patterns of user engagement.

**Figure 2.**
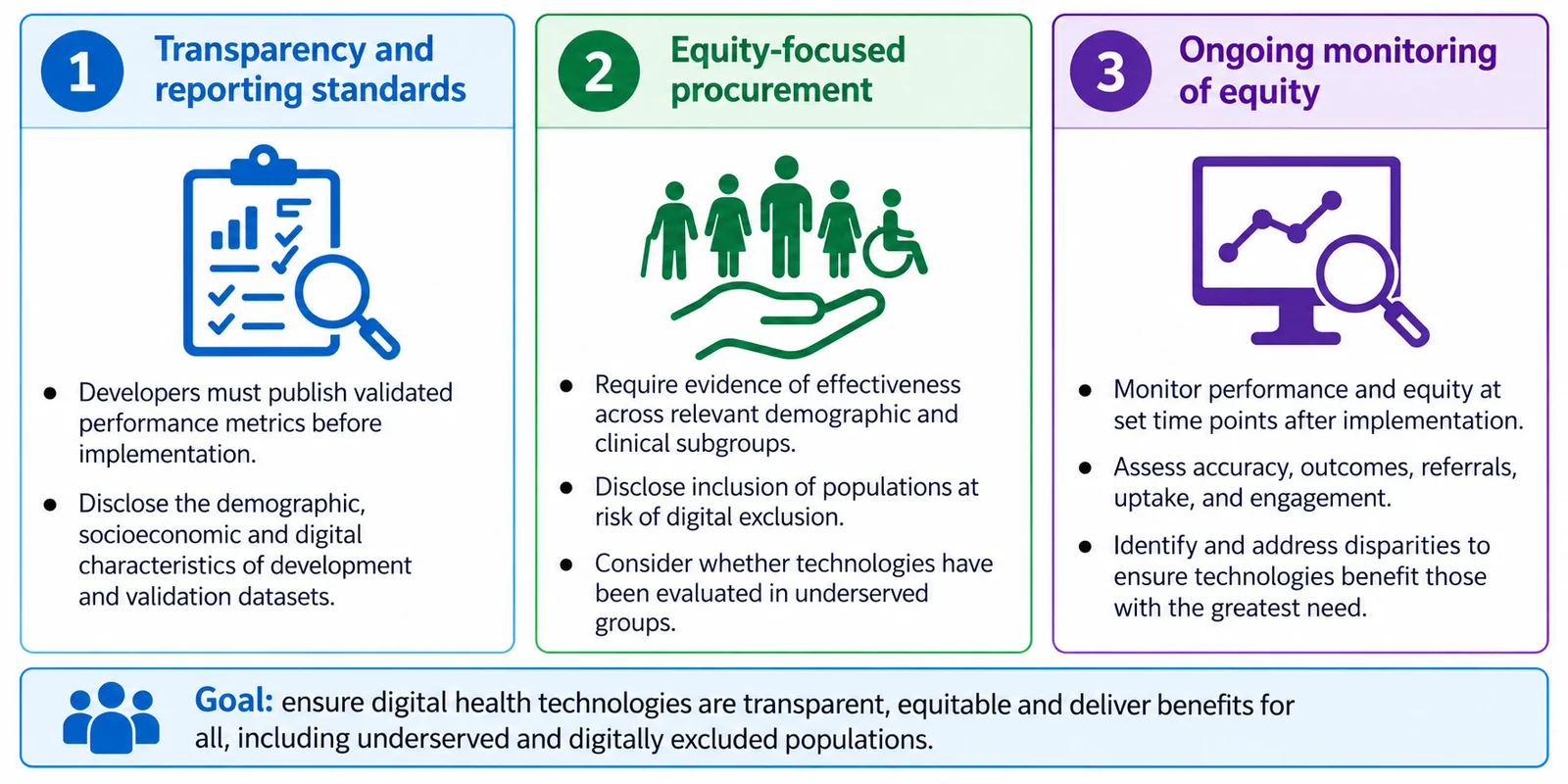
Summary of recommendations for reframing accountability in commercial digital health development and deployment.

This approach of monitoring and evaluation would enable health care organizations to identify whether technologies disproportionately benefit populations that are already digitally engaged while failing to reach those with the greatest health and social care needs. Monitoring should therefore evaluate both algorithmic fairness and implementation of equity to ensure that digital health technologies deliver equitable benefits in routine clinical practice. Figure 2 depicts a new framework for reframing accountability in commercial digital health development and deployment.

## Conclusions

Within the high-income health care settings predominantly represented in the current evidence base, health care organizations should prioritize measures to ensure that AI systems are fair, transparent, and equitable. Current discussions often frame algorithmic fairness as a technical challenge for data scientists. However, responsibility for equitable performance should extend across the digital health ecosystem. Commercial developers are responsible for generating representative evidence, regulators for scrutinizing subgroup performance, health care organizations for considering equity during procurement, and health systems for monitoring real-world impacts after implementation. Without clearly assigned responsibilities, concerns regarding fairness risk remain aspirational rather than operational. Where innovation leaves particular groups behind, it undermines not only health inequity but also the evidentiary foundations of clinical practice. Ensuring that commercial digital health technologies meet standards of generalizability commensurate with the diversity of high-income patient populations is therefore not aspirational but essential to safe, effective, and ethically defensible health care in the digital era.

## Abbreviations

SMI: serious mental illness

## References

[1] Yeung AW, Torkamani A, Butte AJ (2023). The promise of digital healthcare technologies. Front Public Health.

[2] Chinta SV, Wang Z, Palikhe A (2025). AI-driven healthcare: fairness in AI healthcare: a survey. PLOS Digit Health.

[3] Norori N, Hu Q, Aellen FM, Faraci FD, Tzovara A (2021). Addressing bias in big data and AI for health care: a call for open science. Patterns (N Y).

[4] Liu M, Ning Y, Teixayavong S (2023). A translational perspective towards clinical AI fairness. NPJ Digit Med.

[5] Chin MH, Afsar-Manesh N, Bierman AS (2023). Guiding principles to address the impact of algorithm bias on racial and ethnic disparities in health and health care. JAMA Netw Open.

[6] Sjoding MW, Dickson RP, Iwashyna TJ, Gay SE, Valley TS (2020). Racial bias in pulse oximetry measurement. N Engl J Med.

[7] Pfohl SR, Foryciarz A, Shah NH (2021). An empirical characterization of fair machine learning for clinical risk prediction. J Biomed Inform.

[8] Obermeyer Z, Powers B, Vogeli C, Mullainathan S (2019). Dissecting racial bias in an algorithm used to manage the health of populations. Science.

[9] Seyyed-Kalantari L, Zhang H, McDermott MB, Chen IY, Ghassemi M (2021). Underdiagnosis bias of artificial intelligence algorithms applied to chest radiographs in under-served patient populations. Nat Med.

[10] Adamson AS, Smith A (2018). Machine learning and health care disparities in dermatology. JAMA Dermatol.

[11] Crossley S, Baybutt M (2024). Access to healthcare: underserved communities. Int J Health Promot Educ.

[12] Gianfrancesco MA, Tamang S, Yazdany J, Schmajuk G (2018). Potential biases in machine learning algorithms using electronic health record data. JAMA Intern Med.

[13] Rajkomar A, Hardt M, Howell MD, Corrado G, Chin MH (2018). Ensuring fairness in machine learning to advance health equity. Ann Intern Med.

[14] Smith L, Yousef Y, Wasilewski P, Dambha-Miller H (2026). When markets shape AI mental health self-management tools: consequences for serious mental illness. JMIR Ment Health.

[15] Yao R, Zhang W, Evans R, Cao G, Rui T, Shen L (2022). Inequities in health care services caused by the adoption of digital health technologies: scoping review. J Med Internet Res.

[16] (2019). Exploring the UK’s digital divide. Office for National Statistics.

[17] Badr J, Motulsky A, Denis JL (2024). Digital health technologies and inequalities: a scoping review of potential impacts and policy recommendations. Health Policy.

[18] (2024). Regulation (EU) 2024/1689 of the European Parliament and of the Council of 13 June 2024 laying down harmonised rules on artificial intelligence and amending regulations (EC) no 300/2008, (EU) no 167/2013, (EU) no 168/2013, (EU) 2018/858, (EU) 2018/1139 and (EU) 2019/2144 and directives 2014/90/EU, (EU) 2016/797 and (EU) 2020/1828 (Artificial Intelligence Act). European Union.

[19] (2021). Ethics and governance of artificial intelligence for health: WHO guidance. World Health Organization.

[20] Evidence standards framework for digital health technologies. National Institute for Health and Care Excellence.

[21] (2020). A buyer’s guide to artificial intelligence in health and care. NHS England.

